# Supporting rigor through reproducibility

**DOI:** 10.1093/jamiaopen/ooaa050

**Published:** 2020-10-28

**Authors:** Indra Neil Sarkar

**Affiliations:** o1 Rhode Island Quality Institute, Providence, Rhode Island, USA; o2 Center for Biomedical Informatics, Brown University, Providence, Rhode Island, USA

A fundamental tenet of biomedical and health research is the design of studies that are rigorously designed. One way that this rigor is evaluated is through peer-review. The editorial team, along with many peer-reviewers from the community, work hard to assess the work described in manuscripts submitted to *JAMIA Open*. As we continue to refine our processes to improve the core reviewing and editorial processes, we are looking at the next major innovations that will further support the best exposition of the highest quality science.

The peer-review process can be slow and is one of the most difficult aspects that we can control in terms of time to review completion. We continue to explore ways to improve the speed to reviewer selection and guide manuscripts through the review process. Each of you who has authored a manuscript understands, firsthand, the frustration felt when there are delays in reviews. I therefore ask that, when asked to review a manuscript for *JAMIA Open*, that you respond as soon as you can—even if you cannot perform a review; a decline to review is better than no response, and is the bane of most peer-review processes. If you agree to review a manuscript, it is equally important that your review is submitted as close to the requested deadline as possible.

Community abstracts are now mandatory for accepted submissions to *JAMIA Open.* Community abstracts support a key goal in dissemination of published work to the stakeholders that have the most to gain– the patients. I acknowledge the challenge for many of us who spend our time writing and communicating to research audiences to write Community Abstracts. However, for our field to have the most impact, we must convey our work to the greater community. Community understanding for our findings and innovations in leveraging informatics approaches to improve health and health care is a crucial first step toward building a foundation for reproducibility. That is, if a patient understands work presented in a publication, they should expect that it can be reproduced for themselves.

Reproducibility is on equal footing with rigor in terms of importance in the pursuit of knowledge. Many funding agencies now require explicit description for how proposed work will not only be rigorous but also reproducible. *JAMIA Open* has strongly encouraged the availability of any associated data (eg, through Dryad) to support reproducibility. Data that are made available through readily accessible, public repositories supports not only verification studies, but can also form the basis for new studies. Data sets can also be enhanced and curated to provide common “benchmark” datasets for algorithm evaluation.

*JAMIA Open* was established as a Level 1 Data Availability journal, meaning that authors were encouraged to share their data publicly. We are now shifting to be a Level 2 Data Availability journal, meaning that, in addition to sharing data publicly, each publication must include a Data Availability Statement. Of course, the release of data should only be done where ethically possible and in accordance to relevant laws. A description of the Oxford University Press Data Availability policies can be found here: https://academic.oup.com/journals/pages/authors/preparing_your_manuscript/research-data-policy.

Along with data, many of the studies reported in *JAMIA Open* include software. There is encouragement for authors to make their code available through an open-source license. In *JAMIA Open’*s vision to present work as “living” or “dynamic” publications, I would like to now further underscore the value of all authors to submit code using open (eg, in a public repository like GitHub). Furthermore, I encourage the use of resources like Code Ocean or Jupyter Notebooks to share live versions of their code. For now, these live aspects of publications can be linked from manuscripts. An eventual goal will be to develop ways to integrate live content directly into publications. As we evolve our data availability policies, we will concurrently be looking at similar policies for code availability that will further enhance the reproducibility of the published work in *JAMIA Open*.

As *JAMIA Open* continues to grow in readership and presence in the biomedical and health research communities, I hope that these data and code policies will further demonstrate the high-quality of the studies that are selected for publication. Please continue to share your favorite articles on social media, and please call out those papers that do an exceptional job with sharing their data and code. Science is only made better with each of us supporting the rigorous process that ensures reproducibility.

